# *OTC* gene duplication as the possible cause of massive hyperammonaemia with a fatal prognosis

**DOI:** 10.1016/j.ymgmr.2024.101146

**Published:** 2024-09-28

**Authors:** Borkowska Natalia, Kaluzny Lukasz, Rokicki Dariusz, Szmida Elzbieta, Kowalski Pawel, Dus-Zuchowska Monika, Skiba Pawel, Ciara Elzbieta, Biela Mateusz, Rydzanicz Malgorzata, Ploski Rafal, Smigiel Robert

**Affiliations:** a Department of Pediatrics SPZOZ Krotoszyn, Krotoszyn, Poland; b Department of Genetics, Wroclaw Medical University, Wroclaw, Poland; c Department of Pediatric Gastroenterology and Metabolic Diseases, Poznan University of Medical Sciences, Poland; d Department of Pediatrics and Metabolic Pediatrics, Children's Memorial Health Institute, Warsaw, Poland; e Department of Medical Genetics, Children's Memorial Health Institute, Warsaw, Poland; f Department of Diagnostic Imaging, The Children's Memorial Health Institute, Warsaw, Poland; g Department of Pediatrics, Endocrinology, Diabetology and Metabolic Diseases, Wroclaw Medical University, Wroclaw, Poland; h Department of Genetics, Warsaw Medical University, Warsaw, Poland

**Keywords:** *OTC* gene, OTC deficiency, Duplication, Hyperammonaemia

## Abstract

Ornithine transcarbamylase (OTC) deficiency is the most common urea cycle disorder. It may occur due to various changes to the *OTC* gene located on the X chromosome. Many sequence variants in the *OTC* gene result in different severity and require different types of molecular testing. We present a familial case of hyperammonemia possibly caused by the small CNV (duplication) within exon 2 of the *OTC* gene that was not detected by standard sequencing methods. In this case, the knowledge of the underlying molecular changes to the gene results in an appropriate approach to future sibling screening. Collecting more data, especially regarding rare variants of genetic disorders, is essential as it will help to create the best diagnostic-therapeutic path in prenatal and neonatal care in the future. Early diagnosis and treatment can lead to a better prognosis, and this case emphasizes the importance of understanding genetic changes in OTC deficiency.

## 1 Introduction

The ornithine transcarbamylase (OTC; EC 2.1.3.3, OMIM 300461) gene product is a crucial enzyme that plays an essential role in the urea cycle by catalyzing the formation of citrulline from ornithine and carbamyl phosphate in mitochondria of periportal hepatocytes and epithelial cells of the small intestine [1]. OTC deficiency is the most commonly diagnosed urea cycle disorder and occurs in about 1:70,000 births [1]. The gene is located on the X chromosome (Xp11.4). Due to its X-linked inheritance, males tend to be more severely affected, while the phenotype in females varies depending on X-inactivation (from asymptomatic to severe hyperammonemia) [2,3]. Most cases of ornithine transcarbamylase deficiency (OTCD; MIM 311250) are caused by sequence variants in the *OTC* gene that lead to either reduced or absent functional OTC enzyme. Copy number variants can also cause OCTD [[4], [5], [6]] and lead to changes in *OTC* gene expression. The reduction of OTC enzymes leads to a limited ammonia flux through the urea cycle. This results in the accumulation of blood ammonia, which is highly neurotoxic and may manifest as respiratory alkalosis, lethargy, vomiting as well as behavioral and neurological abnormalities, and, in severe cases, coma and death [7,8].

## 2 Cases description

### 2.1 First case

**Fig. 1:**
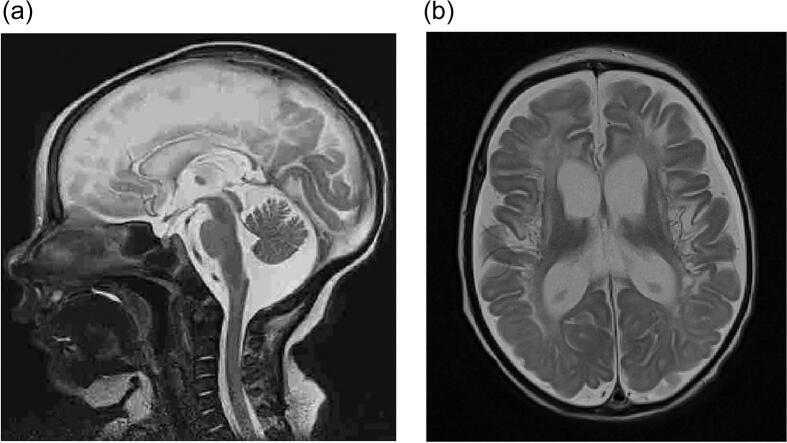
a MRI on day 8 of life - picture of diffuse cytotoxic edema, no signs of bleeding. b MRI at five weeks of age generalized atrophic changes in both brain hemispheres. Broad cisterns of the basal of the brain. T1 -T1-weighted image shows abnormal high signal intensity in frontal and parietal lobes, central furrows, subcortical islands, and basal ganglia. The white matter of both brain hemispheres has decreased volume and abnormal signal intensity, hyperintense on T2-weighted images, and hypointense on T1-weighted images, with cavities forming.

A male infant, known as the proband, was born at 40 weeks of gestational age as a second child of unrelated parents and weighed 3360 g, with an Apgar score of 10 points. From the third day of life, the infant experienced feeding difficulties, muscular hypertonicity, opisthotonos, and respiratory alkalosis. On the fourth day of life, his general condition deteriorated, and he became unresponsive. Due to his symptoms and elevated blood ammonia level (over 2500 μg/dL), a metabolic disorder was suspected. On the same day, he was transferred to a reference hospital for metabolic disorders in children. Upon admission to the hospital, his blood ammonia level was 2617 μg/dL. It was decided to restrict protein in his diet. Pharmacological treatment involving phenylbutyrate, sodium benzoate, and intravenous and oral arginine was started, and continuous veno-venous hemodiafiltration (CVVHDF) was applied. The clinical course and laboratory studies results indicated a deficit of ornithine transcarbamylase (OTC). Biochemical studies showed hyperammonaemia (up to 6639 μg/dL), elevated orotic acid in the urine, very low levels of citrulline in the blood (5,9 umol/L), and elevated plasma glutamine level (2851,6 μmol/L). Despite intensive therapy, the central nervous system suffered severe and irreversible damage. Brain MRI revealed generalized atrophic changes in both brain hemispheres and cystic encephalopathy resulting from a previous cytotoxic brain edema (Fig. 1a-b).

The child was qualified for palliative care and remained in a chronically severe condition; no circulatory or respiratory failure was diagnosed. The neurological examination showed muscular hypertonicity with marked asymmetry, opisthotonos, upper limbs in internal rotation, and microcephaly. Symptomatic treatment was continued: protein-restricted diet, arginine/citrulline, and sodium phenylbutyrate were given via the enteral route. In the following months, the patient required hospitalization to prevent decompensation. Diagnostics were further extended by GC/MS (Gas Chromatography/Mass Spectrometry) examination of the organic acid profile in urine - orotic aciduria was confirmed. At six months of age, during the subsequent hospitalization due to pneumonia, clinical deterioration occurred - increasing drowsiness leading to coma and clinical features of multiple organ failure. The child died on the seventh day of hospitalization after severe deterioration of general condition.

### 2.2 Second case

The female sibling of the proband, the child of the fourth pregnancy, third delivery, was born on time by vaginal labor. The birth weight was 3960 g, and the Apgar score was 10 points. A prenatal diagnosis was conducted, and targeted genetic test for OTC gene was performed. The child was in good general condition during the perinatal period and had ammonia levels within a normal range; the concentration of orotic acid in the urine was within the upper limit of normal. However, in the fourth week of life, the child was admitted to the metabolic clinic due to vomiting.

A physical examination revealed only signs of atopy. Biochemical studies showed elevated serum ammonia concentration (247 μg/dL). At this point, protein was excluded from the diet. Intravenous phenylbutyrate and sodium benzoate were administrated. As a result of treatment, the patient's serum ammonia concentration decreased to 175 μg/dL. The diagnostics were later extended by examining the profile of organic acids in urine using the GC/MS method, showing orotic aciduria, but plasma aminogram and the acylcarnitine profile in a dry blood drop using the tandem mass spectrometry method did not reveal any abnormalities.

At the age of four, the patient experienced a deterioration caused by two decompensation episodes accompanied by hyperammonemia and liver failure. Presently, the patient meets the criteria for liver transplantation.

## 3 Genetic investigations

An NGS (Next Generation Sequencing) metabolic disorders panel was performed to screen for genetic variations at the nucleotide level and failed to detect any disease-causing sequence variant. Next, genetic diagnostics were performed using whole exome sequencing (WES). The library was executed using SeqCap EZ MedExome (Roche, Basel, Switzerland) according to the manufacturer's instruction, and pair-end sequenced (2x100bp) on HiSeq 1500 (Illumina, San Diego, CA, USA) to the mean depth 73×, 98.6 % of the target was covered ≥10×, and 95.9 % covered ≥20×. Bioinformatics analysis of raw WES data and variants prioritization were performed as previously described^10^. WES study also showed no pathogenic or likely pathogenic sequence variants.

**Fig. 2:**
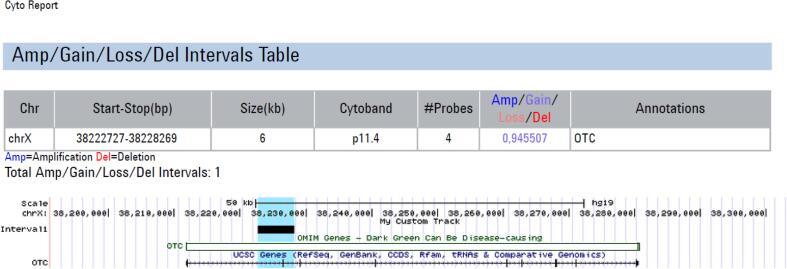
aCGH result arr[GRCh37] Xp11.4(38222727_38228269)x2 and affected gene.

Afterward, an array Comparative Genomic Hybridization (aCGH) study was performed using a microarray platform: Agilent SurePrint G3 CGH ISCA v2, 8x60K (International Standards for Cytogenomic Arrays), which focuses on about 500 regions in the human genome of high clinical significance, and average coverage of the background is about 60 kb. The microarray is based on the genome UCSC hg19 (NCBI Build 37, February 2009). Array-CGH analysis showed an unbalanced profile with an interstitial duplication of approximately 5,5 kbp part of the *OTC* gene (band 11.4, arr[GRCh37] Xp11.4(38222727_38228269)x2) (Fig. 2). The duplicated region contains at least exon 2 of the *OTC* gene.

**Fig. 3:**
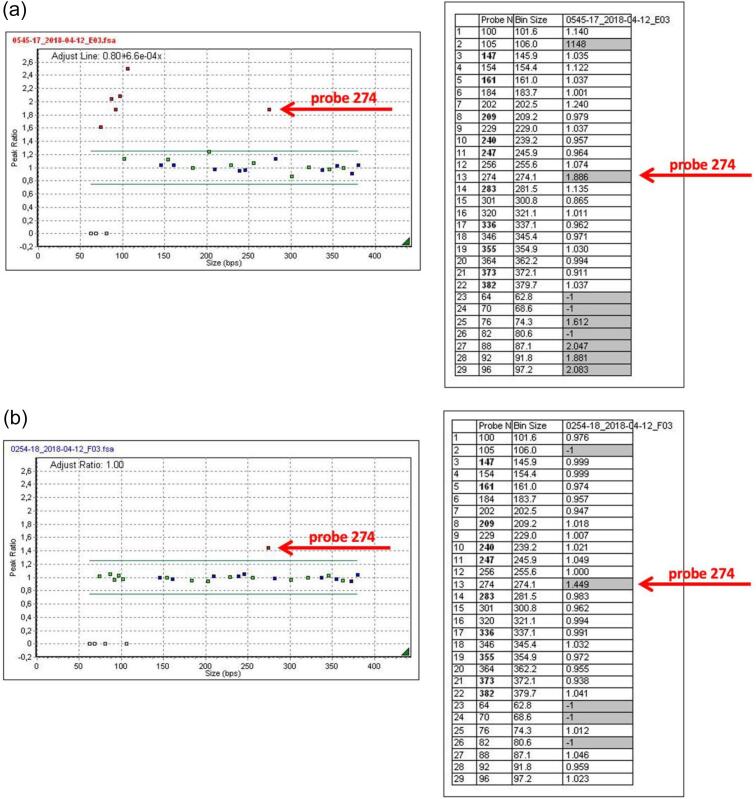
a MLPA P079-A3 OTC proband result. b MLPA P079-A3 OTC mother's result.

The result of aCGH was confirmed by MLPA P079-A3 OTC, which shows increased ligation of probe 274 (02633-L02100) complementary to a sequence fragment in exon 2 of the *OTC* gene (Fig. 3a). This duplication was inherited from apparently healthy mother (Fig. 3b). Moreover, MLPA P079-A3 OTC was used for the prenatal diagnosis in the subsequent pregnancy.

## 4 Discussion

Sequence variant or copy number variation (CNV, mainly deletions) can be identified in 85–90 % of patients with the biochemical phenotype of OTCD [8]. Deletions of the OTC gene are pathogenic. Duplication of the entire *OTC* gene can be a normal variant in the population [4]. However, partial duplication can be disease-causing. Only a few cases of duplication as a possible cause of OTCD have been described [5,6,9,10]. This can occur through two possible mechanisms: chromothripsis, which is caused by chromosome shuttering followed by reunion, and chromoanasynthesis, which is a replication-based mechanism also known as fork stalling and template switching (FoSTeS)/microhomology-mediated break-induced replication (MMBIR). Through an abnormal gene structure, both mechanisms can result in the loss of its function or decreased activity. Upon reviewing the latest literature, a noticeable trend emerges. Over the past few years, a notable increase in OTCD diagnoses coexisting with partial gene duplication has occurred. The course of the disease in the presented family with X-linked OTCD suggests that duplication of the *OTC* gene can be a reason for severe symptoms in both sexes.

We want to emphasize the importance of understanding genetic changes in OTC deficiency. MLPA P079-A3 OTC and aCGH are valuable methods for molecular diagnosis of patients with symptoms of OTCD and negative OTC sequencing. Those methods may be helpful in testing among families affected with OTCD with unknown OTCD causes. In instances where the genetic variant is identified, subsequent pregnancies can be closely monitored and screened for non-CNV sequence variants.

Moreover, knowing how many unidentified cases are attributable to sequence variants detectable through MLPA P079-A3 OTC and aCGH techniques can establish new benchmarks in prenatal screening protocols for families affected by OTCD. Particularly in X-linked diseases, the combined knowledge of the infant's sex and the inheritance status of the sequence variant can guide a potentially lifesaving or health-preserving strategy for female newborns susceptible to developing symptoms in the future.

## CRediT authorship contribution statement

**Borkowska Natalia:** Writing – original draft. **Kaluzny Lukasz:** Supervision. **Rokicki Dariusz:** Supervision. **Szmida Elzbieta:** Supervision. **Kowalski Pawel:** Resources, Data curation, Conceptualization. **Dus-Zuchowska Monika:** Validation, Investigation. **Skiba Pawel:** Resources, Investigation, Data curation. **Ciara Elzbieta:** Resources, Investigation. **Biela Mateusz:** Supervision. **Rydzanicz Malgorzata:** Supervision. **Ploski Rafal:** Supervision. **Smigiel Robert:** Writing – review & editing, Supervision, Conceptualization.

## Declaration of competing interest

The authors declare no competing interests.

## Data Availability

Data will be made available on request.
